# Air Pollution’s Impact on the Economic, Social, Medical, and Industrial Injury Environments in China

**DOI:** 10.3390/healthcare9030261

**Published:** 2021-03-01

**Authors:** Zhong Fang, Pei-Ying Wu, Yi-Nuo Lin, Tzu-Han Chang, Yung-ho Chiu

**Affiliations:** 1 School of Economics, Fujian Normal University, Fuzhou 350007, China; fazhong02@fjnu.edu.cn; 2 Department of Applied Foreign Languages, Cheng Shiu University, 840, Chengcing Rd., Niaosong District, Kaohsiung City 83347, Taiwan; 3 Department of Economics, Soochow University 56, Kueiyang St., Sec. 1, Taipei 10048, Taiwan; 08451081@scu.edu.tw (Y.-N.L.); angleyc06@gmail.com (T.-H.C.); echiu@scu.edu.tw (Y.-h.C.)

**Keywords:** medical treatment, work injury, air pollution, environmental efficiency, meta-DDF

## Abstract

In this era of rapid economic development, it is inevitable that economic activities eventually cause serious damage to the environment’s air quality, making it the focus of global public health. If the treatment efficiency of medical accidents can be improved, then this can significantly stabilize society and improve production efficiency. Past research has mainly focused on work safety and health issues, seldom discussing economic, social, medical, and environmental pollution issues together, and, most generally, adopted static methods that fail to recognize how air pollution affects the overall economy, society, medical care, and external environment. In order to more deeply understand the changes among social, economic activities, and environmental issues due to air pollution, this study proposes a meta-two-stage undesirable dynamic DDF (Direction Distance Function) that, under an exogenous model, divides the 30 provinces of China into high-income regions and middle-income regions and explores the economic, social, medical, and environmental efficiencies between the two areas to resolve the lack of related static analyses. The empirical results are as follows. (1) The AQI (air quality index) significantly impacts the efficiency of medical injuries in various regions. (2) When the AQI is considered, the medical insurance expenditure efficiency score value of high-income areas is lower than the value without the AQI. (3) When the AQI is considered, the efficiency value of the number of work injury insurance benefits in the middle-income area is lower than the efficiency value without the AQI.

## 1. Introduction

The impact of ambient air quality on human health has become the focus of global public health. In regards to China, not only will it restrict the country’s labor development and economic progress, but it may also expose the country’s current medical insurance system to public health shocks caused by air pollution. As residents pay more and more attention to their surrounding ecological environment, the current situation of environmental governance and social insurance in China has become more noticeable.

Poor air conditions are affecting the normal life and physical and mental health of hundreds of millions of people around the world. Air pollution problems lead to various types of diseases, which lead to a decline in the quality of life and physical fitness of residents, which subsequently leads to medical injury problems. According to a report issued by The Global Burden of Diseases (GBD), in 2016, the number of global deaths caused by air pollution was as high as 5.63 million to 6.6 million, and the number of injuries and disability was as high as 150 million to 180 million (Gakidou et al. [1]). Many studies have confirmed that air pollution can cause serious respiratory; cardiovascular; and central nervous system diseases, including ischemic heart disease, stroke, lung cancer, chronic obstructive pulmonary disease, respiratory infections, autism, and hyperactivity, which can even cause fetal malformations (Cohen et al. [2], Lim et al. [3], and Apte et al. [4]). A study using satellite remote sensing images to observe and infer global PM2.5 concentration changes showed that China has poor air governance. Between 1998 and 2012, China’s PM2.5 concentration increased significantly, reaching 2.4 μg per year (Van Donkelaar et al. [5]).

Air pollution poses a serious threat to the health of Chinese residents and also brings huge social health costs to the public and social medical insurance. In recent years, there have been more and more kinds of diseases caused by air pollution in China, including respiratory diseases, cerebrovascular diseases, and so on, and the death toll is also on the rise. A 2017 study confirmed in China that average sulfur dioxide emissions per second will increase the number of deaths caused by lung cancer and respiratory diseases by 0.217 and 1.543, respectively (Chen et al. [6]). According to the China Statistical Yearbook, among the main causes of death for urban residents in some regions in 2019, heart disease accounted for 23.65%, cerebrovascular 20.61%, and respiratory disease 10.36%, ranking second, third, and fourth behind only the number of deaths from malignant tumors. This public health crisis caused by the living environment is occurring on a grand scale, impacting a great number of people, and is cross-generational. If the decline in residents’ health cannot be contained, then it will further limit the development of China’s human resources and also cause a heavy burden on the social medical insurance system.

Public health risks caused by air pollution have therefore become the focus of the China government’s attention. Ambient air quality standards, revised for the third time in 2016, now include PM2.5 in the air quality assessment system for the first time. Moreover, the ambient air quality index (AQI) helps provide health guidelines to the public. These measures show the Chinese government’s strong determination to improve the ecological environment and protect human health. The current AQI covers observations of pollutants such as sulfur dioxide, nitrogen dioxide, PM10, carbon monoxide, ozone, and PM2.5. The larger the index value is, the more serious is the pollution. For example, when the AQI is between 0 and 50, the air quality is excellent; when the AQI is greater than 300, the air is severely polluted.

Economic, social, and health problems caused by air pollution have continued to appear in recent years. There is no doubt that air pollution is an important factor affecting health. However, some scholars believe that there is not enough evidence to prove the extent to which air pollution can affect health or economic and social problems and argue that the AQI will not directly affect economic and social or residents’ health problems (Currie [7]). For example, Concus et al. [8] thought that the impact of air on residents’ health was mainly realized through the differential exposure levels. Heutel and Ruhm [9] found that air pollution can indirectly lead to the increase of resident mortality in some areas by affecting the economic level by matching the air pollution data with the regional economic data. Currie [7] believed that environmental pollution will increase the health costs of residents to a certain extent, and pollution sources and the spread of pollutants will cause various indirect effects on the human body, causing damage to human health.

From the perspective of economy and society, many early studies have found that people with relatively low social and economic status are more likely to be exposed to air pollution than people with high social and economic status. Therefore, the health status of residents with high social and economic status is more vulnerable to air pollution and other external factors than those with low social and economic status. (Currie Janet et al. [10] and Currie Janet et al. [11]). Patankar et al. [12] used the air pollution index with postcodes to study the effects of the monetized burden of disease caused by air pollution in developing countries. It was found that the main bearers of the monetized burden of health of air pollution in developed countries were residents, but the difference was that developing countries mainly relied on residents from poor families with low income and low social status bear.

Although various economic, social, and health problems may be affected by many factors, it is undeniable that they have a certain correlation with air pollution. For example, environmental quality problems caused by air pollution lead to the decline of the quality of life of residents and then population migration problems or medical and health problems, and so on. Therefore, the impact of air pollution on individuals and society has attracted widespread attention from scholars from all walks of life in recent years. Past studies have discussed the causal relationship between air pollution and the demand for medical insurance from an economic perspective (Chang et al. [13], Pi et al. [14], and Chen and Chen [15]), with some scholars analyzing the effects of air pollution from a medical perspective. However, the former perspective only analyzes the supply and demand of medical insurance from a single viewpoint, while the latter is mostly based on the research of a disease itself. It is difficult for their conclusions to effectively link the economic, social, medical, and environmental pollution issues with macrolevel factors. A reasonable analysis on the efficiency of the social security system under the influence of air pollution is thus warranted.

Based on the above considerations and past studies that mainly discuss work safety and health, we see that the impact of work injuries on workers in different occupations has not been evaluated together with the economic, social, medical, and environmental pollution issues. Moreover, the methods typically used are static and regression analyses, which ignore dynamic changes. Therefore, in order to fill the gap in the literature, this research adopted the meta-two-stage undesirable dynamic DDF (Direction Distance Function) under an exogenous model, which takes into account the variations of different regions and the influences of the external environment.

This study offers three contributions. First, it explores economic, social, and environmental efficiencies and also analyzes the impact of government investment in work-related injuries and medical welfare insurance on occupational labor efficiency. Second, it compares the influence of economic and social efficiencies under exogenous environmental conditions. Third, it considers dynamic changes by dividing 30 provinces in China into high-income regions and middle-income regions; discusses the economic, social, medical, and environmental efficiencies of each province; and divides them into two stages: production stage and social insurance treatment stage. The aim is to obtain a more objective efficiency evaluation and to better understand the current status of air pollution prevention and control and social insurance implementation in China, so as to rigorously quantify the environmental costs and potential risks to public health, which can assist in formulating environmental policies and optimizing social security. 

## 2. Literature Review

The past literature on social and medical work injuries is divided into three main directions. One strand is data envelopment analysis (DEA) research on social insurance. The second direction presents the possible impacts of air quality on social human capital. The third one covers the causal relationship between air pollution and the demand for insurance from an economic perspective. The literature review runs as follows.

For a DEA study of social insurance, Wei and Liang [16] employed per capita pension and welfare benefits, per capita social security subsidies, and per capita retirement expenses of administrative institutions as inputs and then utilized pension insurance coverage, unemployment insurance coverage, and medical insurance. The results were that the operating efficiency of social security funds is at a low level. Luo and Chai [17] used the CCA-DEA model to evaluate the efficiency of China’s social security. Their results showed that old-age insurance, medical insurance, unemployment insurance, work-related injury insurance, and both maternity insurance and social welfare are all efficient. Habibov and Fan [18] applied the window DEA model. The input items of provincial expenditures for social welfare programs and market income of the provincial population and the output item of poverty reduction effectiveness measures are used to compare the performances of provincial jurisdictional social welfare programs in poverty reduction. Wang and Liu [19] utilized local government health expenditures as input items and the number of health institutions, the number of health technicians, and the number of beds in health institutions as output items. They analyzed the efficiency of local public health expenditures in China, showing a waste of 24% in health expenditures. He and Li [20] used per capita GDP and total financial expenditure as input items and financial expenditure on social security, percentage of financial expenditure on social security, and per capita financial expenditure on social security as output items to evaluate China’s social security efficiency.

Yu [21] employed the three-stage DEA model to evaluate the efficiency of China’s safe guard expenditure. The results showed that environmental factors have a significant impact on the efficiency of social security expenditures. Moreno and Lozano [22] explored the efficiency of public financial management in European countries to analyze the phenomenon of overspending, unreasonable debt, excessive taxation, and maintaining the level of social welfare under the financial crisis by using a two-stage network DEA model. The results were that both high-deficit countries and, also, major countries such as Germany and France performed poorly. Sui and Yang [23] utilized per capita social security expenditure as the input item and the number of social services and facilities, elderly population pension beds, hospital beds, and the Engel coefficient as output items. Hu et al. [24] applied the three-stage DEA model to analyze the efficiency of the social security system in 29 provinces of China, showing the results of per capita GDP, urbanization rate, marketization rate, and financial autonomy that have a significant impact on efficiency.

The second strand of the literature involves the possible impact of air quality on human capital. Graff Zivin and Neidell [25] found that, when the ozone content is far below federal air quality standards, it has a significant impact on productivity. Lavy et al. [26] found that air pollution will decrease the productivity of jobs that require a high degree of mental concentration. Hanna and Oliva [27] took the closure of a large oil refinery as a study event and found that a 19.7% reduction in sulfur dioxide pollution can increase the working hours of residents in the surrounding area by 1.3 h. Herrnstadt and Muehlegger [28] determined the causal relationship between air pollution and the crime rate.

Aragón et al. [29] found that moderate levels of pollutants reduce the working hours of adults, in turn cutting down on the labor supply. Chang et al. [30] noted that an increase in PM2.5 concentration leads to a significant decrease in productivity, while pollutants such as ozone that do not spread indoors have little effect on productivity. Chen et al. [31] found that air pollution can cause health problems for students and reduce school attendance. The impact lasts at least four days. Chen et al. [32] found that, for every increase of one standard deviation in PM 2.5 concentration, the probability of severe mental illness increases by 6.67%, which affects men and the elderly. Dechezleprêtre et al. [33] found that air pollution leads to a reduction of market economic activities in the entire economy. He et al. [34] confirmed that air pollution has a lagging negative effect on labor productivity. Their study showed that if PM 2.5 increases by 10 μg/m^3^; then, the output drops by 1% after 25 days. Zivin et al. [35] found that fires reduce the total score of the college entrance examination, thereby reducing the possibility of being promoted to first-tier universities.

The third strand of the literature presents the causal relationship between air pollution and the demand for insurance from an economic perspective. Chang et al. [13] noted that every time the air pollution level increases by one standard deviation, the number of insurance contracts signed on that day increases by 7.2%. Pi et al. [14] surveyed elderly people in 23 provinces in China and found that the degree of air pollution has changed. The worse one’s self-assessed health status is, the fewer opportunities there are to purchase medical insurance. Chen and Chen [15] used China’s Health and Nutrition Survey data to investigate the causality of air pollution on the demand for medical insurance. Their results presented that the impact of air pollution on the demand for medical insurance is mainly reflected in women, children, the elderly, high incomes, and education. Zhao [36] used the logit and Poisson regression models to study the financial situation of Chinese households in 2013 and 2015, finding that air pollution significantly increases the possibility of households buying insurance and the level of premium expenditures.

The previous literature has focused on medical insurance and endowment insurance when discussing social insurance, but few studies have included the most relevant work-related injury insurance for young and middle-aged labor in a social security system. Fang et al. [37] discussed the impact of air pollution on the efficiency of the social security system. However, whether this impact will change due to differences in local economies is critical and unclear. Therefore, on the basis of Fang et al. [37], this present study divided China’s provinces into high-income regions and middle-income regions and developed a meta-two-stage undesirable dynamic DDF under an exogenous model in order to explore the local economy’s impact on the model. At the same time, this study added bad outputs in the first and second stages to reflect the shortcomings of the social security system.

## 3. Method

### 3.1. Introduction to the DEA Model

DDF (Direction Distance Function) is a commonly used efficiency measurement tool, because it can deal with input reduction and increase output at the same time. Chung et al. [38] put forward the concept of a distance function based on output orientation, which is an extended orientation output distance function (RDF). The traditional DDF is a radial measurement model, but the efficiency calculation fails to include all non-zero differences and all sources of inefficiency. Therefore, the efficiency value of an institute is overestimated (Feng et al. [39] and Fang et al. [40]). To solve this type of problem, Färe and Grosskopf [41] and Chen et al. [42] established a non-radial direction distance function.

The DDF non-radial distance function has better evaluation performance and can provide more reasonable and accurate estimation results. However, Färe and Grosskopf [41] noted that the DDF non-radial distance function fails to consider two-stage inter-period continuous effects, regional differences, and external environmental issues. Following the Feng et al. [39], Fang et al. [40], Li et al. [43], and Tone and Tsutsui [44] dynamics, two-stage DEA, and O’Donnell et al. [45] common boundary (meta-frontier), this paper proposes a meta-two-stage undesirable dynamic DDF under an exogenous model. This present paper uses this model to evaluate production efficiency and social insurance treatment efficiency in China to avoid the underestimation or overestimation of efficiency values.

### 3.2. Meta-Two-Stage Undesirable Dynamic DDF under an Exogenous Model

We assume that, due to different management types, resources, regulations, or environments, all manufacturers (N) are composed of decision-making units (DMUs) (N = N1 + N2 + … + Ng) of g groups. Suppose there are two stages (with corresponding efficiency) in each *t* time period $\left(t=1,\ldots ,T\right)$: production efficiency and social insurance treatment efficiency. The production stage has m inputs $\;x_{ij}^{t}\left(i=1,\ldots ,m\right)$ to generate D intermediate products $z_{dj}^{t}\left(d=1,\ldots ,D\right)$ and K desirable outputs $q_{kj}^{t}\left(k=1,\ldots ,K\right)$. The social insurance treatment stage produces a desirable S1 output $y_{rj}^{vt}\left(r=1,\ldots ,s1\right)$ and undesirable S2 output $y_{rj}^{bt}\left(r=1,\ldots ,s2\right)$ from D intermediate products $z_{dj}^{t}\left(d=1,\ldots ,D\right)$ and C inputs $w_{cj}^{t}\left(c=1,\ldots ,C\right)$. Lastly, $c_{hj}^{d^{t-1}}\left(h=1,\ldots ,H\right)$ is a carryover factor, and there are V external variables $b_{uj}^{t}\left(U=1,\cdots V\right)$. Under the meta-frontier, the decision unit k can choose the final output that is most favorable to its maximum value, and so, the efficiency of DMU_k_ under the common boundary can be solved by the following linear programming procedure.

(a) Objective function

Overall efficiency:

The DMU efficiency is:

(1) $$\max\;\mathrm{Meta}\;\mathrm{frontier}\;\mathrm{efficiency}\left(\mathrm{MFE}\right)=\overset{G}{\underset{g=1}{\sum }}\overset{T}{\underset{t=1}{\sum }}\gamma_{tg}\left(w_{1g}^{t}\theta_{1g}^{t}+w_{2g}^{t}\theta_{2g}^{t}\right)$$

subject to

Production efficiency stage Social insurance treatment efficiency stage

$$\overset{G}{\underset{g=1}{\sum }}\overset{n}{\underset{j}{\sum }}\lambda_{jg}^{t}X_{ijg}^{t}\leq \theta_{1g}^{t}X_{ijg}^{t}\;\forall i\;\forall t\;\overset{G}{\underset{g=1}{\sum }}\overset{n}{\underset{j}{\sum }}\mu_{jg}^{t}Z_{djg}^{t}\leq \theta_{2g}^{t}Z_{djg}^{t}\;\forall d\;\forall t\;$$

$$\overset{G}{\underset{g=1}{\sum }}\overset{n}{\underset{j}{\sum }}\lambda_{jg}^{t}z_{djg}^{t}\leq \theta_{1g}^{t}z_{djg}^{t}\;\forall d\;\forall t\;\overset{G}{\underset{g=1}{\sum }}\overset{n}{\underset{j}{\sum }}\mu_{jg}^{t}y_{rjg}^{vt}\geq \theta_{2g}^{t}y_{rjg}^{vt}\;\forall r\;\forall t\;$$

$$\overset{G}{\underset{g=1}{\sum }}\overset{n}{\underset{j}{\sum }}\lambda_{jg}^{t}q_{kjg}^{t}\geq \theta_{1g}^{t}q_{jg}^{t}\;\forall k\;\forall t\;\overset{G}{\underset{g=1}{\sum }}\overset{n}{\underset{j}{\sum }}\mu_{jg}^{t}y_{rjg}^{bt}\leq \theta_{2g}^{t}y_{rjg}^{bt}\;\forall r\;\forall t$$

$$\overset{G}{\underset{g=1}{\sum }}\overset{n}{\underset{j}{\sum }}\lambda_{jg}^{k}\leq 1\;\forall t\;\;\;\overset{\;G}{\underset{g=1}{\sum }}\overset{n}{\underset{j}{\sum }}\mu_{jg}^{t}w_{cjg}^{t}\leq \theta_{2g}^{t}w_{cjg}^{t}\;\forall c\;\forall t$$

$$\lambda_{j}^{t}\geq 0\;\forall j\;\forall t\;\;\;\overset{G}{\underset{g=1}{\sum }}\overset{n}{\underset{j}{\sum }}\mu_{jg}^{t}=1\;\forall t\;$$

(2) $$\mu_{j}^{t}\geq 0\;\forall j\;\forall t$$

Exogenous variables:

(3) $$\sum_{j=1}^{T}\lambda_{1}^{t}b_{Uj}^{t}=\theta_{1}^{t}b_{U}^{t}\;\forall U\;\forall t$$

Link of the two stages:

$$\overset{G}{\underset{g=1}{\sum }}\overset{n}{\underset{j=1}{\sum }}\lambda_{jg}^{t}Z_{djg}^{t}=\overset{G}{\underset{g=1}{\sum }}\overset{n}{\underset{j=1}{\sum }}\mu_{jg}^{t}Z_{djg}^{t}\;\;\forall d\;\forall t$$

Link of the two periods:

(4) $$\overset{G}{\underset{g=1}{\sum }}\overset{n}{\underset{j=1}{\sum }}\lambda_{jg}^{t-1}c_{hjg}^{t}=\overset{G}{\underset{g=1}{\sum }}\overset{n}{\underset{j=1}{\sum }}\lambda_{jg}^{t}c_{hjg}^{t}\;\;\forall h\;\forall t$$

Among them, $\gamma_{t}$ is the weight assigned to period *t*, and $w_{1}^{t}$ and $w_{2}^{t}$ are the weights assigned to the production efficiency stage and social insurance treatment efficiency stage in time period *t*, respectively. Therefore, for each time period *t*, $w_{1}^{t},\;w_{2}^{t},\;\gamma_{t}\geq 1,\;\mathrm{and}\;\overset{G}{\underset{g=1}{\sum }}\overset{T}{\underset{t=1}{\sum }}\gamma_{tg}=1$.

We calculated the following three efficiency groups through linear programming. 

(1) Stage efficiency: 

Stage 1: Production efficiency value

The efficiency of stage L (L = 1, 2) of the DMU to be evaluated is appraised relative to each period *t* $\left(t=1,\ldots ,T\right)$. Stage efficiency can be expressed as:

(5) $$\rho_{1}^{t^{*}}=1-\theta_{l}^{t^{*}};\;l=\;1,2;\;t=1,2,\cdots ,T$$

Stage 2: Social insurance treatment efficiency value

(6) $$\rho_{2}^{t^{*}}=1-\sum_{t=1}^{T}\gamma_{t}\theta_{t}^{t^{*}};\;l=\;1,2$$

(2) Period efficiency value:

In this group, the overall efficiency of each period *t* of the DMU being evaluated is

(7) $$\rho^{t^{*}}=w_{1}^{t}\rho_{1}^{t^{*}}+w_{2}^{t}\rho_{2}^{t^{*}};\;t=1,2,\cdots ,T$$

(3) Overall efficiency:

In this group, the overall efficiency of the DMU being evaluated is assessed. The overall efficiency is given by the weighted sum of periodic efficiency on *t*, shown as:

(8) $$\rho^{*}=\sum_{t=1}^{T}\gamma_{t}\rho^{t^{*}}$$

### 3.3. Group-Frontier Efficiency (GFE)

As each DMU under the group frontier chooses the most favorable final weighted output, the DMU efficiencies under the group frontier are solved using the following equations:

(a) Objective function

The DMU efficiency is

(9) $$\max\;\mathrm{Group}\;\mathrm{frontier}\;\mathrm{efficiency}\;\left(\mathrm{GFE}\right)=\overset{T}{\underset{t=1}{\sum }}\gamma_{t}\left(w_{1}^{t}\theta_{1}^{t}+w_{2}^{t}\theta_{2}^{t}\right)$$

subject to

Production efficiency stage Social insurance treatment efficiency stage

$$\overset{n}{\underset{j}{\sum }}\lambda_{j}^{t}X_{ij}^{t}\leq \theta_{1}^{t}X_{ij}^{t}\;\forall i\;\forall t\;\;\overset{n}{\underset{j}{\sum }}\mu_{j}^{t}Z_{dj}^{t}\leq \theta_{2}^{t}Z_{dj}^{t}\;\forall d\;\forall t\;$$

$$\overset{n}{\underset{j}{\sum }}\lambda_{j}^{t}z_{dj}^{t}\leq \theta_{1}^{t}z_{dj}^{t}\;\forall d\;\forall t\;\;\;\;\;\;\;\;\;\;\overset{n}{\underset{j}{\sum }}\mu_{j}^{t}y_{rj}^{vt}\geq \theta_{2}^{t}y_{rj}^{vt}\;\forall r\;\forall t\;\;$$

$$\overset{n}{\underset{j}{\sum }}\lambda_{j}^{t}q_{kj}^{t}\geq \theta_{1}^{t}q_{kj}^{t}\;\forall k\;\forall t\;\;\;\;\;\;\;\;\;\;\overset{n}{\underset{j}{\sum }}\mu_{j}^{t}y_{rj}^{bt}\leq \theta_{2}^{t}y_{rj}^{bt}\;\forall r\;\forall t\;$$

$$\overset{n}{\underset{j}{\sum }}\lambda_{j}^{k}\leq 1\;\forall t\;\;\;\;\;\;\;\;\;\;\;\;\;\;\;\;\;\;\;\;\overset{n}{\underset{j}{\sum }}\mu_{j}^{t}w_{cj}^{t}\leq \theta_{2}^{t}w_{cj}^{t}\;\forall c\;\forall t\;$$

$$\lambda_{j}^{t}\geq 0\;\forall j\;\forall t\;\;\;\overset{n}{\underset{j}{\sum }}\mu_{j}^{t}=1\;\forall t\;$$

(10) $$\;\mu_{j}^{t}\geq 0\;\forall j\;\forall t$$

Exogenous variables:

(11) $$\sum_{j=1}^{T}\lambda_{1}^{t}b_{Uj}^{t}=\theta_{1}^{t}b_{U}^{t}\;\forall U\;\forall t$$

Link of the two stages:

$$\overset{n}{\underset{j=1}{\sum }}\lambda_{j}^{t}Z_{dj}^{t}=\overset{n}{\underset{j=1}{\sum }}\mu_{j}^{t}Z_{dj}^{t}\;\;\forall d\;\forall t$$

Link of the two periods

(12) $$\overset{n}{\underset{j=1}{\sum }}\lambda_{j}^{t-1}c_{hj}^{t}=\overset{n}{\underset{j=1}{\sum }}\lambda_{j}^{t}c_{hj}^{t}\;\;\forall h\;\forall t$$

Among them, $\gamma_{t}$ is the weight assigned to period *t*, and $w_{1}^{t}$ and $w_{2}^{t}$ are the weights assigned to the production efficiency stage and social insurance treatment efficiency stage in time period *t*, respectively. Therefore, for each time period *t*, $w_{1}^{t},\;w_{2}^{t},\;\gamma_{t}\geq 1,\;\mathrm{and}\;\overset{T}{\underset{t=1}{\sum }}\gamma_{t}=1$.

We calculate the following three efficiency groups through linear programming. 

(1) Stage efficiency: 

Stage 1: Production efficiency value 

In this group, the efficiency of stage L (L = 1, 2) of the DMU to be evaluated is gauged relative to each period *t* $\left(t=1,\ldots ,T\right)$. The stage efficiency is expressed as

(13) $$\rho_{1}^{tg}=1-\theta_{l}^{tg^{*}};\;l=\;1,2;\;t=1,2,\cdots ,T$$

Stage 2: Social insurance treatment efficiency value

(14) $$\rho_{2}^{tg}=1-\sum_{t=1}^{T}\gamma_{t}\theta_{t}^{tg^{*}};\;l=\;1,2$$

(2) Period efficiency value:

In this group, the overall efficiency of each period t of the DMU being evaluated can be expressed as

(15) $$\rho^{tg}=w_{1}^{t}\rho_{1}^{tg}+w_{2}^{t}\rho_{2}^{tg};\;t=1,2,\cdots ,T$$

(3) Overall efficiency:

In this group, the overall efficiency of the DMU being evaluated is assessed. The overall efficiency is given by the weighted sum of periodic efficiency on *t*, shown as

(16) $$\rho_{}^{*g}=\overset{T}{\underset{t=1}{\sum }}\gamma_{t}\rho^{tg}$$

### 3.4. Technology Gap Ratio (TGR)

As the meta-frontier model contains g groups, the technical efficiency of the meta-frontier efficiency (MFE) is less than the technical efficiency of the group frontier efficiency (GFE). The ratio value, or the technology gap ratio (TGR), is

(17) $$\mathrm{TGR}=\frac{\rho^{*}}{\rho_{}^{*g}}=\frac{MFE}{GFE}$$

### 3.5. Input, Desirable Output, and Undesirable Output Efficiencies

We utilized Hu and Wang’s [45] total-factor energy efficiency index to overcome any possible biases in the traditional efficiency indicators, for which there are eight key efficiency factors: Employed population, Number of work injuries, Work injury insurance expenditure, Medical insurance expenditure as inputs, GDP, Number of invalid deaths, and Work injury insurance benefits as outputs and Fixed assets as the carryover variable. Here, “I” represents area, and “*t*” represents time. The efficiency models are defined as follows.

(18) $$\mathrm{Input}\;\mathrm{efficiency}\;=\;\frac{\mathrm{Target}\;\mathrm{input}}{\mathrm{Actual}\;\mathrm{input}}$$

(19) $$\mathrm{Undesirable}\;\mathrm{output}\;\mathrm{efficiency}\;=\;\frac{\mathrm{Target}\;\mathrm{Undesirable}\;\mathrm{output}}{\mathrm{Actual}\;\mathrm{Undesirable}\;\mathrm{output}}$$

(20) $$\mathrm{Desirable}\;\mathrm{output}\;\mathrm{efficiency}\;=\;\frac{\mathrm{Actual}\;\mathrm{Desirable}\;\mathrm{output}}{\mathrm{Target}\;\mathrm{Desirable}\;\mathrm{output}}$$

If the target inputs and undesirable output equal the actual inputs and undesirable output, then the efficiencies are 1, which indicates an overall efficiency. However, if the target inputs and undesirable output are less than the actual inputs and undesirable output, then the efficiencies are less than 1, which indicates an overall inefficiency.

If the target desirable outputs are equal to the actual desirable outputs, then the efficiencies are 1, indicating an overall efficiency. However, if the target desirable outputs are more than the actual desirable outputs, then the efficiencies are less than 1, indicating an overall inefficiency.

## 4. Empirical Study

### 4.1. Data and Variables

This study collected data from China Statistical Yearbook, China Environmental Statistical Yearbook, and China Labor Statistics Yearbook from 2013 to 2017 and used panel data to conduct empirical research on 30 provinces, autonomous regions, and municipalities in China (ex-Tibet). Among them, the AQI pollutant data came from the annual report of the China Environmental Protection Agency. The Chinese government has started to systematically count data on air pollution since 2013. On the other hand, some provinces have not counted statistics on medical injuries since 2017. Considering data validity, this study selected the time span from 2013 to 2017. The various variables used in the study appear in Table 1 below.

First stage: Production stage
Input variables:

(A) Employed population (unit: 10,000) is the number of urban labor registrations in each region at the end of each year.

Output variables:

(B) GDP (unit: 100 million RMB) covers the regional GDP of each province, municipality, and autonomous region based on the current level.

Link between Production stage and Social insurance treatment stage:

(C) Number of work-related injuries (unit: person) covers the number of casualties in various regions that are directly or indirectly caused by work.

Second stage: Social insurance treatment stage
Input variables:

(D) Work injury insurance expenditure (unit: 100 million RMB) is calculated from the basic fund expenditure of work injury insurance in each region.

(E) Medical insurance expenditure (unit: 100 million RMB) comes from the basic fund expenditure of urban basic medical insurance in various regions.

Output variables:

(F) Work injury insurance benefits (unit: 10,000 million RMB) represent the number of compensation projects and standards that the relatives of workers who have suffered work-related injuries and died in various regions shall be entitled to, according to the law.

(G) Number of invalid deaths (unit: person) comes from people who died of a sudden illness during working hours and/or at work or who died within 48 h after emergency treatment. They are deemed as work-related injuries in each region.

Carryover:

(H) Fixed assets (unit: RMB million) are calculated according to all fixed asset investments in each region, including domestic investment; Hong Kong, Macao, and Taiwan investment; foreign investment; real estate investment; etc.

Exogenous:

(I) AQI (air quality index) is derived from measuring the concentrations of pollutants and describes levels of clean or polluted air and health effects. Pollution monitoring includes particulate matter (PM2.5 and PM10), sulfur dioxide (SO2), ozone (O3), nitrogen dioxide, and carbon monoxide (PM2.5 and PM10 are 24-h average concentrations).

**Figure 1:**
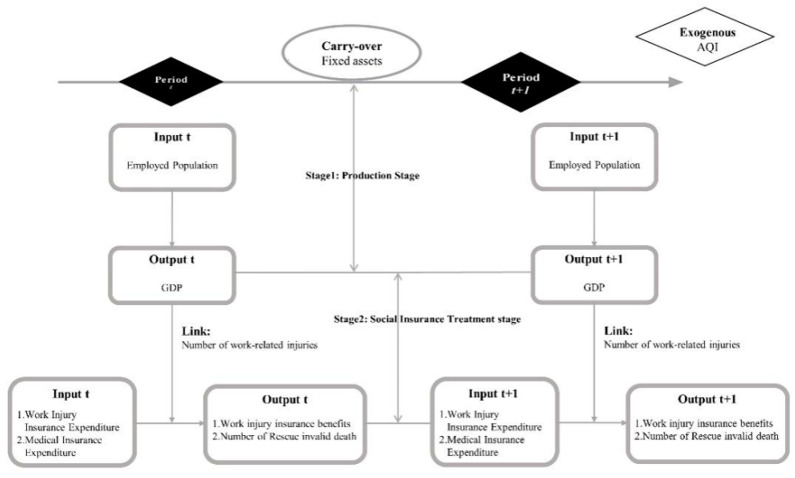
Model framework.

We use the modified undesired two-stage Dynamic DDF model, the AQI as an exogenous variable, and the number of work injuries as an intermediate output variable to analyze the existence of estimation bias in an efficiency analysis. Based on these assumptions, we designed a meta-two-stage undesirable dynamic DDF under an exogenous model (see Figure 1); used it to study the relationships among economic, social, medical, and environmental pollution problems in the 30 provinces, autonomous regions, and municipalities of China; evaluated the medical work injury efficiency; and put forward corresponding policy suggestions. The first stage is the production stage, where the input variable is employed population and the output variable is GDP, and then, the second stage is imported through the link variable of injury count. The second stage is the social insurance treatment stage, with industrial injury insurance and health insurance as inputs, industrial and commercial insurance as outputs, and the carryover as fixed investments.

### 4.2. Descriptive Statistics of Relevant Indicators such as Inputs and Outputs

**Figure 2:**
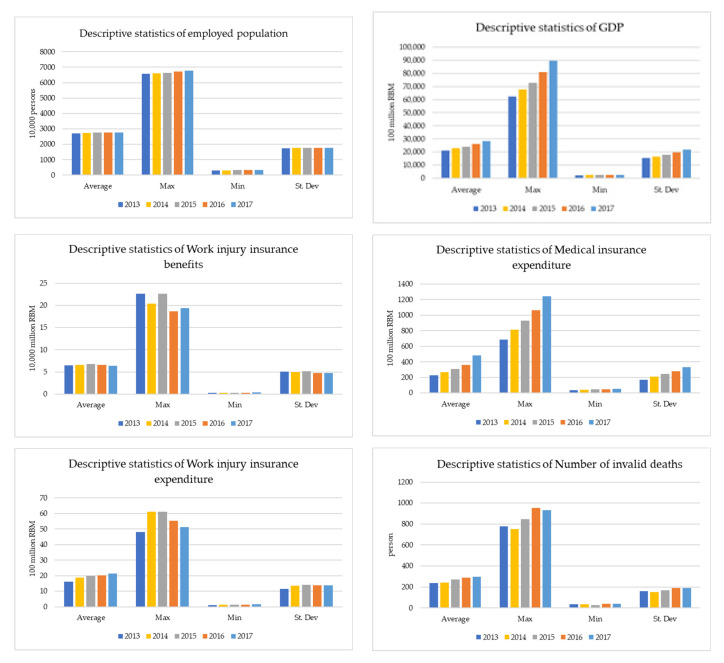
Input–output statistics (2013–2017).

Figure 2 shows the input and output indicators, including the input of employed population, fixed assets, and the output of GDP in the production phase. It also presents the results of statistical analysis on the expenditures of industrial injury insurance and medical insurance during the period of social insurance (2013–2017).

From 2013 to 2017, the maximum and minimum values of employed population (input) increased slowly, mainly due to the disappearance of China’s demographic dividend, the slow growth of its population, and the gradual decline of the employed population. From the input of fixed assets, the average value and the maximum value increased year by year, and the minimum value increased in all other years, except for a small decrease in 2016. The GDP, its maximum value, and its average value also showed a slow growth trend, while the minimum value’s growth is slow and slightly fluctuating. The maximum value of industrial injury insurance expenditure fluctuated somewhat and was higher in 2014 than in 2013 but has been decreasing year by year since 2015. Its average value continues to rise. From 2013 to 2017, the average value, maximum value, and minimum value of medical insurance expenditure increased continuously, and the difference between the maximum value and minimum value has become larger and larger, indicating that the medical insurance expenditure maintained a certain growth trend. From the number of workers’ injury insurance benefits, the maximum value fluctuated from 2014 to 2013 and began to rise in 2015. In 2016, it fell and reached a new low. In 2017, it rose slightly to be higher than that in 2016 but lower than in 2014. Its minimum and the mean have fluctuated slightly. The average number of invalid deaths has been increasing year by year, but the maximum fluctuated and decreased in 2014, which is lower than the number of invalid deaths in 2013. However, the number of invalid deaths started to rise in 2015, continued to rise to a new level in 2016, and decreased slightly in 2017. The minimum reached a new low in 2015 but increased for the rest of the years.

This paper discusses the input–output index statistics of China’s 30 provinces by dividing them into high-income regions and middle-income regions according to their economic development levels. The high-income region (Group 1) includes Beijing, Shanghai, Fujian, Guangdong, Hubei, Hunan, Jiangsu, Liaoning, Inner Mongolia, Shandong, Shaanxi, Tianjin, Zhejiang, and Chongqing. The middle-income region (Group 2) is Gansu, Guangxi, Guizhou, Hainan, Hebei, Henan, Heilongjiang, Jilin, Jiangxi, Ningxia, Qinghai, Shanxi, Xinjiang, and Yunnan.

Table 2 shows a comparison of the input–output indicators between the two income regions from 2013 to 2017. We see that the average labor input of high-income regions is significantly greater than that of middle-income regions, growing slowly in both regions from 2013 to 2016 and declining slightly in 2017. The average GDP of high-income regions is more than that of middle-income regions, and the GDP of high-income regions continues to increase greatly, while the GDP of middle-income regions also continues to increase but at a slower rate. Overall, the gap between the average GDP in high-income regions and that in middle-income regions has widened. The average fixed asset investment in high-income regions is also greater than that in middle-income regions, and both averages show a continuous upward trend. The average number of work injuries is higher in the high-income regions than in the middle-income regions, and the average number of work injuries fell in both regions from 2013 to 2016 but rose slightly in both regions in 2017. According to the regional average AQI Index, the difference between high-income and middle-income regions is very small. The average AQI emissions in both regions have continued to decline to a minimum by 2017.

### 4.3. Comparative Analysis of the Total Factor Efficiency in the Regions

**Figure 3:**
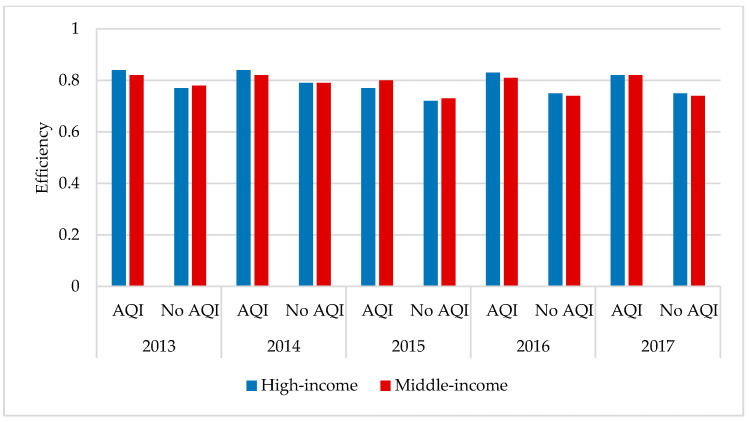
Total efficiency from 2013–2017 between the high- and middle-income regions. AQI: air quality index.

As seen from Figure 3, in the case of the AQI, the average overall efficiency score of the high-income regions was higher than that of the middle-income regions in 2013, 2014, and 2016, while the overall efficiency score of the high-income regions was lower than that of the middle-income regions in 2015 and flat in 2017. The average efficiency scores of both the high-income and middle-income regions showed some fluctuations, meaning that there is still more room for improvement in both regions. Without taking into account the AQI, the middle-income region efficiency score showed a slight increase in the average overall efficiency score from 0.77 in 2013 to 0.78 in 2014, a significant decrease to 0.72 in 2015, and a recovery in 2016 and 2017, but it remained at 0.74. Similarly, the trend of change in the average overall efficiency score between the high-income and middle-income regions was similar, with a final decrease after the fluctuations from 0.77 in 2013 to 0.75 in 2017.

In both high- and middle-income regions, the efficiency scores were higher between 2013 and 2017 when the AQI was considered than when the AQI was not considered. The average total efficiency score of the high-income regions is higher than that of the middle-income regions, which shows that the former is better than the latter in air pollution prevention and treatment. However, the high-income regions’ efficiency has suppressed the tendency for the efficiency to decline. In contrast, the middle-income regions’ efficiency scores are generally stable and do not fluctuate significantly, indicating a relatively limited room for improvement.

### 4.4. Comparative Analysis of the TGR in the Regions

**Figure 4:**
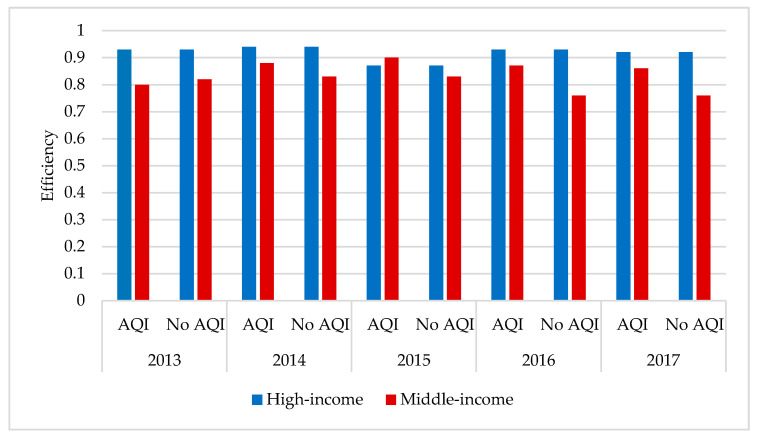
Table technology gap from 2013-2017 between the high- and middle-income regions.

Figure 4 shows that, when considering the AQI, there is a certain gap between the technology differential rate of high-income regions and that of middle-income regions. However, the gap is not very large, and the high-income region technology difference rate is more than 0.9. In 2015, the high-income province AQI values were lower than middle-income provinces, but the gap was the smallest. On the whole, the high-income areas are ahead of the middle-high-income areas, but there is still a lot of room for improvement. Thus, the authorities should take measures to strengthen their governance, and more intensive governance should be implemented in the middle- and high-income regions.

### 4.5. Comparative Analysis of the Efficiency Values of the Input Indicators for the Regions

Table 3 shows the average efficiency scores of the input indicators, such as employment, Industrial Injury Insurance Expenditure, and medical insurance expenditure between 2013 and 2017.

In the case of the AQI, the average efficiency of employment input for four years in the high-income regions was higher than that in the middle-income regions, but the average efficiency of employment input in the high-income provinces showed a downward trend. The average score of the middle-income provinces was 0.95 over five years and showed some fluctuations. The room for improvement was slightly higher than that in high-income provinces. When the AQI was not taken into account, the five-year average efficiency of labor input in the high-income regions was higher than in the middle-income regions for the remaining four years, except for 2014, when it was slightly lower than in the middle-income regions. In terms of the average input efficiency of the labor force, the trends of the high-income regions and the middle-income regions were consistent, rising continuously in the first three years and decreasing in 2016 and 2017.

When considering the AQI, with the exception of 2016, the high-income regions scored higher on the labor input efficiency than without the AQI. Only in 2013 and 2017 were labor inputs more efficient in the medium-sized regions than without the AQI. Moreover, the average labor input efficiency score of the high-income regions was higher than that of the middle-income regions, showing that the former is better than the latter in labor force number. However, the efficiency in high-income regions fluctuated, from 1 in 2013 to 0.87 in 2016, and recovered in 2017 but still has not reached the level before the decline. Some measures need to be taken to stabilize the current efficiency and arrest the decline in efficiency. The labor force efficiency score for the middle-income regions fluctuated less between 2013 and 2017, but by a small margin, indicating the need to maintain labor input.

According to the average efficiency score of industrial injury insurance expenditure, the high-income regions had scores from four years that were greater than those in the middle-income regions when considering the AQI. The average expenditure efficiency of industrial injury insurance in the high-income regions showed a fluctuating trend, from 0.96 in 2013 to 0.92 in 2016. The same indicators for the middle-income provinces were fluctuating and unstable. There is some room for improvement in this index in both types of income-level provinces.

When the AQI was not considered, the difference in efficiency scores between the two groups of regions was not significant, and the indicator for the high-income regions had scores during the four years that were higher than that for the middle-income regions. Only in 2015 was its industrial injury insurance expenditure efficiency slightly lower than the middle-income region’s. The average expenditure efficiency of industrial injury insurance in the high-income regions showed a rising trend, from 0.94 in 2013 to 0.96 in 2017. The index of the industrial injury insurance expenditure efficiency in the middle-income regions also fluctuated, rising from 0.94 in 2013 to 0.95 in 2017.

The average scores of the industrial injury insurance expenditure under the two situations of considering the AQI and not considering the AQI were quite similar. It showed that environmental pollution had little effect on the expenditure of industrial injury insurance in different provinces. This showed that the AQI did not directly affect the expenditure of industrial injury insurance, mainly because the efficiency of industrial injury insurance expenditure was more directly related to the local economic revenue and medical investment, and air pollution was only a driving factor.

In terms of the average efficiency scores for health-care expenditures, the high-income provinces had less than the middle-income provinces when the AQI was considered. The high-income provinces showed a fluctuating downward trend, becoming less than 0.79 in the fifth year. The middle-income provinces also showed a fluctuating downward trend, falling to 0.82 in the fifth year, and so the gap between them and the high-income provinces was not large. There is plenty of room for improvement in both provinces, though. When the AQI was not taken into account, there was no significant difference in the average efficiency score between the two regions, and the index fluctuated over time. However, there is still room for improvement.

The efficiency scores of the health insurance expenditures in the high-income regions from 2013 to 2017 were lower when the AQI was considered than when the AQI was not considered. In contrast, the middle-income regions scored higher on the efficiency scores when considering the AQI than without the AQI. This indicated that high-income regions overestimate the efficiency of AQI health insurance expenditures, while middle-income regions underestimate it.

### 4.6. Comparative Analysis of Efficiency Values of Output Item Indicators in the Regions

Table 4 shows regional classifications based on the high- and middle-income regions from 2013 to 2017. When the AQI was considered, the differences in the average GDP efficiency scores between the two groups were not significant, except for the first year when the median income efficiency was close to 1 at 0.99, whereas, for the other years, it was 1. The results show that the efficiency scores of the two types of regions are good. When the AQI was excluded, the differences in the average GDP efficiency scores between the two groups were not significant, with the high-income regions scoring slightly less than 1, or 0.99, for the GDP efficiencies in 2014 and 2015. The remaining years were all 1, with only the middle-income region scoring slightly below 1 for efficiency in 2015 and only 2014 for the two regions, while the high-income region scored below the middle-income region for GDP efficiency. Generally speaking, the differences in GDP efficiencies between the two regions were not big, and the improvement space is limited. It can be seen that, although the AQI will affect the regional economic development, it does not directly affect the efficiency of GDP in China’s provinces, because regional resource endowment and policy orientation are the direct factors driving the development of regional GDP.

As can be seen from the efficiency score of the number of workers covered by occupational injury insurance, by taking into account the AQI, the high-income regions were slightly higher than the middle-income regions for four years, with a maximum difference of 0.02. One year was slightly lower than the middle-income provinces, with a difference of 0.01. On the whole, the changes in the two types of regions were not big, at no more than 0.05, basically maintaining a relatively stable trend.

The efficiency scores of high-income regions when considering the AQI were all lower in the last three years in the number of workers receiving injury insurance than without considering the AQI. When the AQI was considered, the efficiency scores of the high-income regions showed a fluctuating downward trend from 2013 to 2017, while when the AQI was not considered, the efficiency scores of the region were relatively stable, ranging from 0.99 to 1. This showed that high-income areas need more investment in air pollution treatment and should take on more effective measures. Looking at the efficiency score for the number of workers covered by industrial injury insurance without taking into account the AQI, the efficiency scores were above 0.9 in both the high-income and middle-income regions, and the efficiency scores fluctuated, but there was a small difference between the two types of regional efficiency. The fluctuation of the efficiency score in the middle-income regions was consistent with that in the high-income regions. Overall, the high-income areas need to take measures to curb the decline in efficiency scores in a timely manner, while the middle-income areas have relatively stable efficiency scores but also a relatively large room for improvement. On the other hand, from the score value of the efficiency of the number of industrial injury insurance benefits, we can see that the effect of the AQI on the efficiency of the number of employees with industrial injury insurance is not significant, which shows that the effect of the AQI on the number of employees of industrial injury insurance is indirect, because the efficiency of the number of employees with industrial injury insurance is directly related to the number of employees and the investment of insurance funds.

The average score for the number of ineffectual deaths showed a fluctuating downward trend when considering the AQI, from 0.92 in 2013 to 0.82 in 2017, but the efficiency was still higher than that of the middle-income provinces over a five-year period. The middle-income efficiency score first experienced a decline and then an increase, showing an overall trend of instability, indicating that the middle-income regional performance was not stable enough, and there is a relatively greater room for improvement. The scope for improvement in high-income provinces is also expanding. Excluding the AQI, the average efficiency score for the high-income regions dropped from 0.88 in 2013 to 0.84 in 2017, while the average efficiency score for the middle-income regions dropped from 0.86 in 2013 to 0.81 in 2017. The difference in the average efficiency scores of the two regions is widening, which indicates that the improvement space for the two areas is expanding.

The high-income regions also scored higher than those without AQI from 2013 to 2017 when the AQI was considered, while the middle-income regions scored slightly higher in efficiency, except in 2017, when the AQI was not considered. The remaining four years were lower than without taking into account the AQI. It shows that environmental pollution has a great influence on the efficiency of invalid death numbers and that there are overestimates or underestimates in different regions.

### 4.7. Comparative Analysis of the Efficiency Value of the Number of Industrial Injuries by Regions

**Figure 5:**
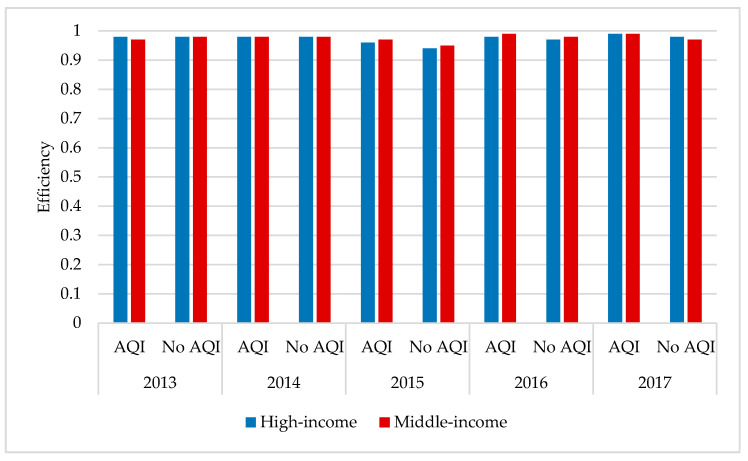
Number of work-related injuries from 2013–2017 between high- and middle-income regions.

According to Figure 5, when considering the AQI, the work injury efficiency of the two regions is greater than 0.9, and both of them are stable. The difference in efficiency values between the two regions is the largest in 2015 but at less than 0.01, indicating that the difference in average efficiency values between the two regions is not significant. In addition, the efficiency of the number of workers injured in high-income areas fluctuates upward, reaching a maximum of 0.99 in 2017. The efficiency of middle-income cities shows the same trend, reaching 0.99 in 2016 and 2017, displaying that the work injury efficiency improved in the two regions.

In the case of the AQI, the efficiency score for the number of work injuries in the high-income regions was higher than the efficiency score for the high-income regions without the AQI for five years. Except for 2013, the middle-income regions also scored higher on efficiency when the AQI was considered than when the AQI was not considered. In both cases, the lowest efficiency scores for the high-income and middle-income regions were in 2015, but they recovered in later years, with the high-income regions rising from 0.93 to 0.99. Taking into account the AQI, the middle-income regions’ efficiency rose from 0.97 to 0.99; without the AQI, the high-income regions’ efficiency rose from 0.93 to 0.97, while the middle-income regions’ efficiency grew from 0.95 to 0.97. The high-income regions had lower efficiency scores than the middle-income regions in 2013, without taking into account the AQI, and were slightly higher than the middle-income regions in 2017, widening the gap in the average efficiency scores between the two groups. The provinces had more room for improvement when the AQI was not considered than when the AQI was considered. Overall, both regions need to take steps to stabilize their current efficiency.

## 5. Conclusions and Policy Recommendation

The control of urban air pollution is related to the physical and mental health of a country’s residents, not only directly affecting their health but also causing an increasing probability of medical work accidents. The conclusions of this research are as follows.

1. In the total factor efficiency analysis, both the high-income and middle-income regions have higher efficiency scores when considering the AQI than that when not considering the AQI. Moreover, the average total efficiency scores of high-income regions are higher than those of middle-income regions, which shows that the former are better than the latter in air pollution prevention and control. However, the high-income regions have a wide range of fluctuations in their scores and show a downward trend, which means there is still room for improvement. There is no significant difference in technological efficiency between the high-income and middle-income provinces when the AQI is considered and when the AQI is not considered. This suggests that air pollution does not have a significant impact on the technical differential efficiency of the provinces, but high-income and middle-income provinces both have large fluctuations in efficiency values, indicating that there is still considerable room for improvement.
2. In terms of the labor input efficiency analysis, there are four years in which the labor input efficiency scores of high-income regions are higher when considering the AQI than when not considering it. Only in 2013 and 2017 are the labor inputs more efficient in the medium-sized regions with the AQI than without the AQI. Moreover, the average scores of the labor force input efficiency in high-income regions are higher than those in middle-income regions, which shows that the former are better than the latter in this efficiency, but the efficiency of the high-income provinces fluctuates. In the efficiency analysis of the health insurance expenditure, the efficiency scores of high-income regions are lower when the AQI is considered than when the AQI is not considered. On the contrary, the efficiency scores in the middle-income regions are higher under the AQI than those without the AQI. High-income regions tend to overestimate the efficiency of health insurance expenditures when the AQI is considered, while middle-income regions tend to underestimate the efficiency of health insurance expenditures. In contrast, environmental pollution has little impact on the cost of work injury insurance in these provinces, and the regional average scores are not significantly different when the AQI is considered.
3. In terms of the efficiency analysis of the number of employees injured by work, when considering the AQI, the efficiency in the middle-income region is lower in the five years versus without considering the AQI, while the high-income region’s efficiency scores in the last three years after considering the AQI arr lower than that when not considering the AQI. This shows that environmental pollution has some influence on the efficiency value of the number of workers injured, and there is still some room for improvement. In the analysis of the number of ineffectual deaths, the efficiency scores of the high-income regions are higher than those of the middle-income regions without the AQI for all five years, while the efficiency scores of the middle-income regions are slightly higher than those without the AQI in 2017. The remaining four years are less than without taking into account the AQI. It shows that environmental pollution has a great influence on the efficiency of invalid death numbers, and there are overestimates or underestimates in different regions. By contrast, the AQI has little impact on the efficiency of injury counts in both regions. There is a similar case for GDP, in that with or without the AQI, the efficiency of GDP is close to or is 1.

Based on the above empirical analysis, we can see that the AQI obviously affects the efficiency of medical injuries in high-income and middle-income regions. Therefore, this paper suggests that governments at all levels in China should make further efforts to optimize the natural environment and reduce air pollution as follows.

1. In order to improve the local air quality, specific environmental policies should be formulated according to the characteristics of different regions. High-income provinces have more advanced pollution treatment technology and less energy-consuming industries, and so, most of the high-income provinces exhibit AQI governance efficiency. However, the characteristics of the industrial structures in most low-income provinces determine that there is still a large number of industries with high energy consumption and high pollution, which seriously restrict the efficiency of air pollution control in these regions. Lagging far behind the high-income regions, low-income areas also have more medical work accidents, and so, these areas should adjust their industrial structure to strengthen the control of environmental pollution at its source. At the same time, middle-income provinces should be encouraged to actively learn from the governance experience and policy measures of high-income provinces to guide and influence the social and individual behaviors of industrial structures and residents’ production and lives. Governments can design and develop measures to promote health and environmental improvements.
2. There is an urgent need to enhance the expenditure efficiency of industrial injury insurance and medical insurance. Air pollution, especially PM2.5, covers a wide range of harmful chemicals that can cause lasting and serious harm to the health of the population. As the resulting diseases often require significant medical input, they are difficult to cure, which, in turn, poses a serious threat to the sustainable development of the country’s workforce. At present, the efficiency of medical investment and the efficiency of industrial and commercial insurance expenditures in all provinces of China, whether high-income or middle-income, are not very high and fluctuate greatly. In particular, provinces such as Guizhou, Xinjiang, and Yunnan have low efficiency values, which call for further improvement and the optimization of their medical care systems. High-income provinces should continue to ensure the extent of industrial injury insurance expenditures, while middle-income provinces need to significantly increase the scope of both these expenditures and medical insurance expenditures in order to effectively address the air pollution caused by disease treatment and to reduce the number of medical accidents.
3. Governments throughout China can actively learn from the more advanced medical security systems of developed countries. High-income provinces should especially take the lead in improving or reforming their existing medical systems and expand the scope of recipients of industrial injury insurance benefits. Doing so can help effectively prevent air pollution brought about by disease risk and medical work accidents, with the end goal of enhancing the efficiency of health investments and medical injury efficiency and providing the best basic protection for the health of all residents.

## Figures and Tables

**Table 1:** Input and output variables.

	Input Variables	Output Variables	Link	Carryover	Exogenous Variable
Stage 1	Employed population	GDP	Number of work-related injuries	Fixed assets	AQI
Stage 2	Work injury insurance expenditure Medical insurance expenditure	Work injury insurance benefits Number of invalid deaths			

Data source: China Statistical Yearbook Database. AQI: air quality index.

**Table 2:** Input and output variables from 2013–2017 between high- and middle-income regions.

Year	Region	Employed Population	GDP	Fixed Assets	Number of Work-Related Injuries	AQI
2013	High-income	3005	23,618	16,173	44,545	126
	middle-income	2836	21,403	15,135	40,586	125
2014	High-income	3036	25,472	18,512	43,070	90
	middle-income	2865	23,085	17,401	39,291	89
2015	High-income	3044	26,935	20,341	40,402	79
	middle-income	2874	24,364	19,137	36,828	78
2016	High-income	3057	29,090	22,008	38,885	77
	middle-income	2888	26,263	20,670	35,559	76
2017	High-income	3055	31,593	23,404	38,939	74
	middle-income	2887	28,545	21,942	35,706	73

**Table 3:** Input variables from 2013–2017 between high- and upper-middle-income regions.

Year	Region	Labor		Work Injury Insurance Expenditure		Medical Insurance Expenditure	
		AQI	No AQI	AQI	No AQI	AQI	No AQI
2013	High-income	1.00	0.96	0.96	0.94	0.89	0.92
	middle-income	0.97	0.96	0.95	0.95	0.95	0.93
2014	high-income	1.00	0.97	0.96	0.97	0.83	0.85
	middle-income	0.95	0.97	0.98	0.97	0.87	0.85
2015	High-income	0.99	0.99	0.93	0.91	0.91	0.92
	middle-income	0.97	0.98	0.91	0.91	0.95	0.94
2016	high-income	0.87	0.98	0.93	0.98	0.85	0.91
	middle-income	0.94	0.98	0.97	0.98	0.95	0.93
2017	High-income	0.96	0.95	0.98	0.96	0.79	0.81
	middle-income	0.95	0.93	0.94	0.95	0.81	0.79

**Table 4:** Output variables from 2013–2017 between the high- and upper-middle-income regions.

Year	Region	GDP		Work Injury Insurance Benefits		Number of Invalid Deaths	
		AQI	No AQI	AQI	No AQI	AQI	No AQI
2013	High-income	1.00	1.00	1.00	0.99	0.93	0.88
	middle-income	0.99	1.00	0.98	0.99	0.85	0.86
2014	high-income	1.00	0.99	1.00	0.99	0.88	0.83
	middle-income	1.00	1.00	0.97	0.99	0.78	0.81
2015	high-income	1.00	0.99	0.98	0.99	0.85	0.81
	middle-income	1.00	0.99	0.99	0.99	0.75	0.78
2016	high-income	1.00	1.00	0.98	0.99	0.90	0.86
	middle-income	1.00	1.00	0.98	0.99	0.81	0.83
2017	high-income	1.00	1.00	0.99	0.99	0.85	0.84
	middle-income	1.00	1.00	0.97	0.99	0.82	0.82

## Data Availability

Not applicable.
